# Estrogen receptor signaling mediates leptin-induced growth of breast cancer cells via autophagy induction

**DOI:** 10.18632/oncotarget.22684

**Published:** 2017-11-25

**Authors:** Pawan Kumar Raut, Dong Young Choi, Sang Hyun Kim, Jin Tae Hong, Taeg Kyu Kwon, Jee Heon Jeong, Pil-Hoon Park

**Affiliations:** ^1^ College of Pharmacy, Yeungnam University, Gyeongsan, Republic of Korea; ^2^ Department of Pharmacology, School of Medicine, Kyungpook National University, Daegu, Republic of Korea; ^3^ College of Pharmacy and Medical Research Center, Chungbuk National University, Cheongju, Republic of Korea; ^4^ Department of Immunology, School of Medicine, Keimyung University, Daegu, Republic of Korea

**Keywords:** apoptosis, autophagy, breast cancer, estrogen receptor, leptin

## Abstract

Leptin, a hormone derived from adipose tissue, promotes growth of cancer cells via multiple mechanisms. Estrogen receptor signaling is also known to stimulate the growth of breast cancer cells. However, the involvement of estrogen receptor signaling in the oncogenic actions of leptin and its underlying mechanisms are not clearly understood. Herein, we investigated mechanisms for estrogen receptor signaling-mediated growth of breast cancer cells, particularly focusing on autophagy, which plays a crucial role in leptin-induced tumor growth. Inhibition of estrogen receptor signaling via gene silencing or treatment with a pharmacological inhibitor (tamoxifen) abolished leptin-induced growth of MCF-7 human breast cancer cells. Interestingly, leptin-induced autophagy activation, determined by up-regulation of autophagy-related genes and autophagosome formation, was also significantly suppressed by inhibiting estrogen receptor signaling. Moreover, inhibition of estrogen receptor markedly prevented leptin-induced activation of AMPK/FoxO3A axis, which plays a crucial role in autophagy induction. Leptin-induced cell cycle progression and Bax down-regulation were also prevented by treatment with tamoxifen. The pivotal roles of estrogen receptor signaling in leptin-induced cell cycle progression, apoptosis suppression, and autophagy induction were further confirmed in MCF-7 tumor xenograft model. Taken together, these results demonstrate that estrogen receptor signaling plays a key role in leptin-induced growth of breast cancer cells via autophagy activation.

## INTRODUCTION

Leptin, a hormone primarily produced by adipose tissue, has been well known to modulate appetite and regulate energy balance through binding with its receptors localized in the hypothalamus [1, 2]. In addition to its critical role in the maintenance of energy homeostasis, there is a growing appreciation that leptin signaling is implicated in the wide spectrum of physiological functions. Malfunction of leptin signaling, such as mutation in either leptin or leptin receptor, leads to fatty liver in association with insulin resistance in mice [3]. Furthermore, leptin treatment prominently reduced hepatic triglyceride accumulation [4] and improved type II diabetes in clinical patients [5], clearly indicating a protective role of leptin against obesity and diabetes. Moreover, leptin administration reverses alcohol-induced lipid accumulation in liver, and leptin deficiency contributes to the pathogenesis of alcoholic fatty liver via modulation of AMPK and STAT3 signaling [6], implying the protective role of leptin from alcoholic liver disease. In contrast to the beneficial metabolic effects, it has been reported that increased plasma leptin levels has the positive correlation with the incidence of various types of cancers [7]. Furthermore, activation of leptin signaling contributes to the development and/or progression of cancer through multiple mechanisms [8–10]. For example, leptin is known to activate diverse signaling cascades, such as the JAK/STAT, MAPK, and PI3 kinase/AKT pathways, most of which are implicated in differentiation and proliferation of cancer cells [10]. Leptin treatment has been also demonstrated to suppress apoptosis [11] and accelerate cell cycle progression through induction of the genes related with cell cycle [12].

In the modulation of breast cancer, although leptin is well known to promote growth of breast cancer cells [13–15], it does not significantly affect growth of estrogen receptor (ER)-negative breast cancer cells [16]. Moreover, pretreatment with tamoxifen, a selective pharmacological inhibitor of ER, was found to suppress leptin-induced tumor growth [17]. Although previous reports clearly indicate the crucial role of ER signaling in leptin-induced growth of breast cancer cells, the underlying molecular mechanisms are still largely unknown.

Autophagy, a self-digestive cellular process, is responsible for the removal of unnecessary or dysfunctional cellular components via formation of an autophagosome, which fuses with the lysosome to sequester and degrade target cytoplasmic components. The autophagic process is composed of multiple stages, including nucleation, cargo recognition and selection, autophagosome formation, vesicle fusion, and autophagosome breakdown, which are coordinately controlled by a number of genes referred as autophagy-related genes (ATGs) [18, 19]. While autophagy was originally reported as a distinct type of cell death, an increasing number of studies have recognized its involvement in the adaptive process of the cells exposed to stressful conditions and dysregulated autophagy leads to a number of pathophysiological conditions, including Alzheimer’s disease, Paget’s disease, and aging [20]. Autophagy induction is prominent under conditions of metabolic stress, such as low nutrient levels or hypoxic conditions [18]. Given that tumor tissues are mostly exposed to stressful conditions, the biological roles of autophagy in cancer modulation have been extensively studied. It has been reported that autophagy is reduced in tumor cells [21] and cancer cells with defective autophagy are associated with increased tumor formation [22], thereby suggesting that autophagy induction would play a suppressive role in tumor formation during the early stage of tumorigenesis. However, in contrast to this notion, accumulating evidence has also shown that autophagosome machinery provides the essential nutrients needed to meet the energy requirements for the growth of cancer cells under metabolic stress conditions [23]. Therefore, autophagy plays a prominent role in sustaining cancer cell survival rather than promoting cell death in the case of established tumors. The exact role of autophagy induction in the modulation of cancer remains controversial and the detailed mechanisms underlying the differential role of autophagy in the regulation of tumor development and growth remain to be further investigated.

Estrogen is involved in various biological responses, in addition to its essential roles in sexual development and the reproductive system. Of the two different types of receptors, namely, the estrogen receptor-α (ERα) and -β (ERβ) [24], activation of ERα signaling leads to the proliferation of breast cancer cells via induction of proliferating cell nuclear antigen (PCNA), and suppression of p53 and p21 [25], whereas ERβ signaling inhibits the growth of breast cancer cells [26, 27]. In addition to binding with its ligand (estrogen), ER can be activated via extracellular growth signaling, such as epidermal growth factor (EGF) and insulin-like growth factor-1 (IGF-1). For instance, upon binding with their receptors, EGF or IGF subsequently activates mitogen activated protein kinase (MAPK), which in turn phosphorylates Ser118 in the activation function-1 (AF-1) domain of ERα, resulting in the conformation change and translocation of ERα, transcription of target genes and proliferation of breast cancer cells [28]. Similar to EGF and IGF, leptin binds to its receptor and activates MAPK signaling, leading to the phosphorylation at Ser118 in the AF-1 domain and ERα activation, indicating that leptin induces the transactivation of the ER, even in the absence of estrogen. In addition, leptin stimulates local production of estrogen by increasing the expression and/or activity of aromatase, an enzyme responsible for the conversion of androgens into estrogens, via STAT3 and ERK signaling [29, 30].

AMP-activated protein kinase (AMPK) has been widely recognized as an energy sensor molecule. Aside from its critical role as a primary regulator of energy and metabolic homeostasis, AMPK signaling has been increasingly recognized to play a role in cancer modulation by complicated manners. Activation of AMPK signaling causes reprogramming of tumor cell metabolism, which confers plasticity to allow cancer cells to survive under metabolic stress and produces optimal growth conditions [31]. For example, AMPK activation increases the proliferation rates of prostate cancer cells, aggressive breast tumors and astrocytic tumors [32–34], whereas inhibition of AMPK activity induces apoptosis, thereby suppressing the proliferation of prostate cancer cells [32]. In contrast, recent studies have also demonstrated that AMPK signaling can also exert tumor suppressive effects. For instance, loss of AMPK activity has been observed in various type of cancer cells [35] and treatment with metformin, a pharmacological activator of AMPK, reduces the incidence of cancers [36]. Therefore, AMPK can act as a tumor suppressor or oncogene depending on cellular context. Forkhead box O3A (FoxO3A), a member of forkhead family of transcription factors, mediates diverse biological responses, including cell cycle modulation, angiogenesis and apoptosis [37]. It has been reported that transcriptional activity and expression of FoxO3A are regulated by AMPK signaling [38] and the AMPK/FoxO3A axis is considered one of the critical targets for cancer treatment [39]. Furthermore, recent studies have demonstrated that the AMPK/FoxO3A axis is implicated in the transcription of the genes related with autophagy [40], implying that the AMPK/FoxO3A pathway is one of the key upstream regulators of autophagy induction.

Based on previous reports, it is well established that higher levels of circulating leptin contribute to the development and/or progression of breast cancer and that ER signaling is implicated in leptin-induced tumor growth. However, the molecular mechanisms whereby ER signaling mediates leptin-induced growth of breast cancer are not clearly understood. It has been recently reported that autophagy induction mediates leptin-induced growth of breast and hepatic cancer cells by suppressing apoptosis [11]. In the present study, we investigated the molecular mechanisms underlying leptin-induced growth of breast cancer cells, with focus on the role of ER signaling in relation to autophagy induction. Herein, we have demonstrated for the first time that ER signaling plays a critical role in leptin-induced autophagy induction in breast cancer cells, which in turn leads to both inhibition of apoptosis and acceleration of cell cycle progression. Moreover, we provided the evidence that autophagy induction by ER signaling is mediated, at least in part, via modulation of the AMPK/FoxO3A axis.

## RESULTS

### Leptin-induced growth of breast cancer cells is mediated via estrogen receptor signaling

**Figure 1:**
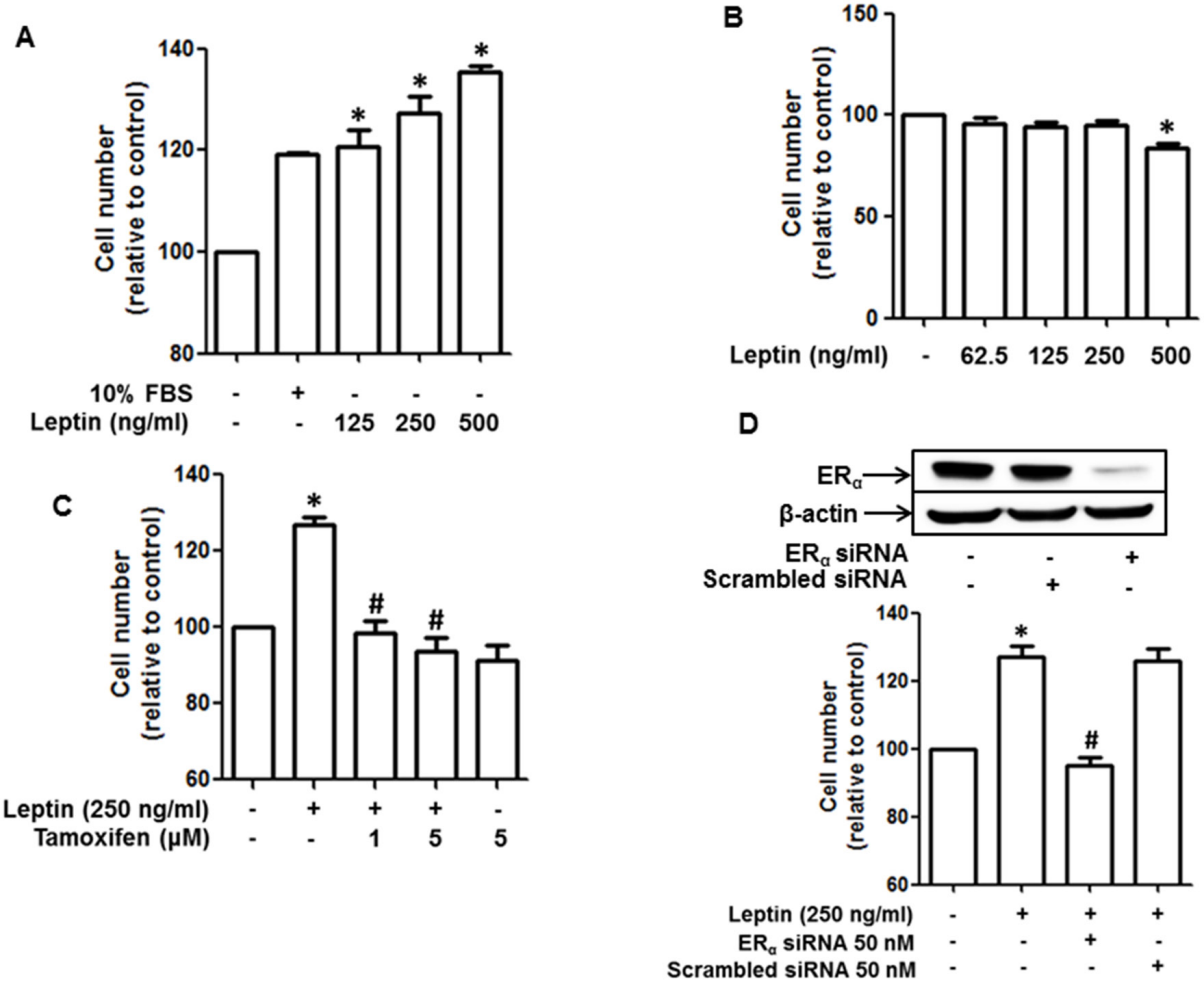
Role of estrogen receptor (ER) signaling in leptin-induced growth of breast cancer cells **(A and B)** MCF-7 breast cancer cells (A) and MDA-MB-231 breast cancer (B) cells were treated with the indicated concentrations of leptin for 48 h. Cell viability was determined by MTS assay as described in the Materials and Methods section. Values are presented as mean ± SEM (n=3). **(C)** MCF-7 cells were pretreated with the indicated concentrations of tamoxifen for 1 h followed by treatment with leptin (250 ng/mL) for an additional 48 h. Cell viability was assessed by MTS assay. Values are presented as mean ± SEM (n=3). **(D)** MCF-7 cells were transfected with 50 nM of siRNA targeting estrogen receptor-α (ERα) or control scrambled siRNA. (Upper panel) Gene silencing efficiency of ERα siRNA was monitored by Western blot analysis. (Lower panel) Cell viability was determined by MTS assay as described previously. In all experiments, ^*^ denotes P < 0.05 compared with control cells and ^#^ denotes P < 0.05 compared with the cells treated with leptin but not treated with tamoxifen or transfected with ERα siRNA.

To elucidate the molecular mechanisms by which estrogen signaling mediates leptin-induced growth in breast cancer cells, we first confirmed the effect of leptin on the growth of breast cancer cells in our experimental conditions. As shown in Figure 1A, leptin treatment significantly promoted the growth of MCF-7 breast cancer cells in a dose-dependent manner, whereas no significant growth effect was observed in MDA-MB-231 cells (Figure 1B), which are ER negative breast cancer cells. Moreover, leptin-induced growth of MCF-7 breast cancer cells was significantly suppressed by pretreatment with tamoxifen (Figure 1C), a selective ER modulator. This result was confirmed by gene silencing of ER-α (Figure 1D). Collectively, these results substantiate the crucial role of ER signaling in leptin-induced growth of breast cancer cells.

### ER signaling is involved in leptin-induced autophagy induction in breast cancer cells

**Figure 2:**
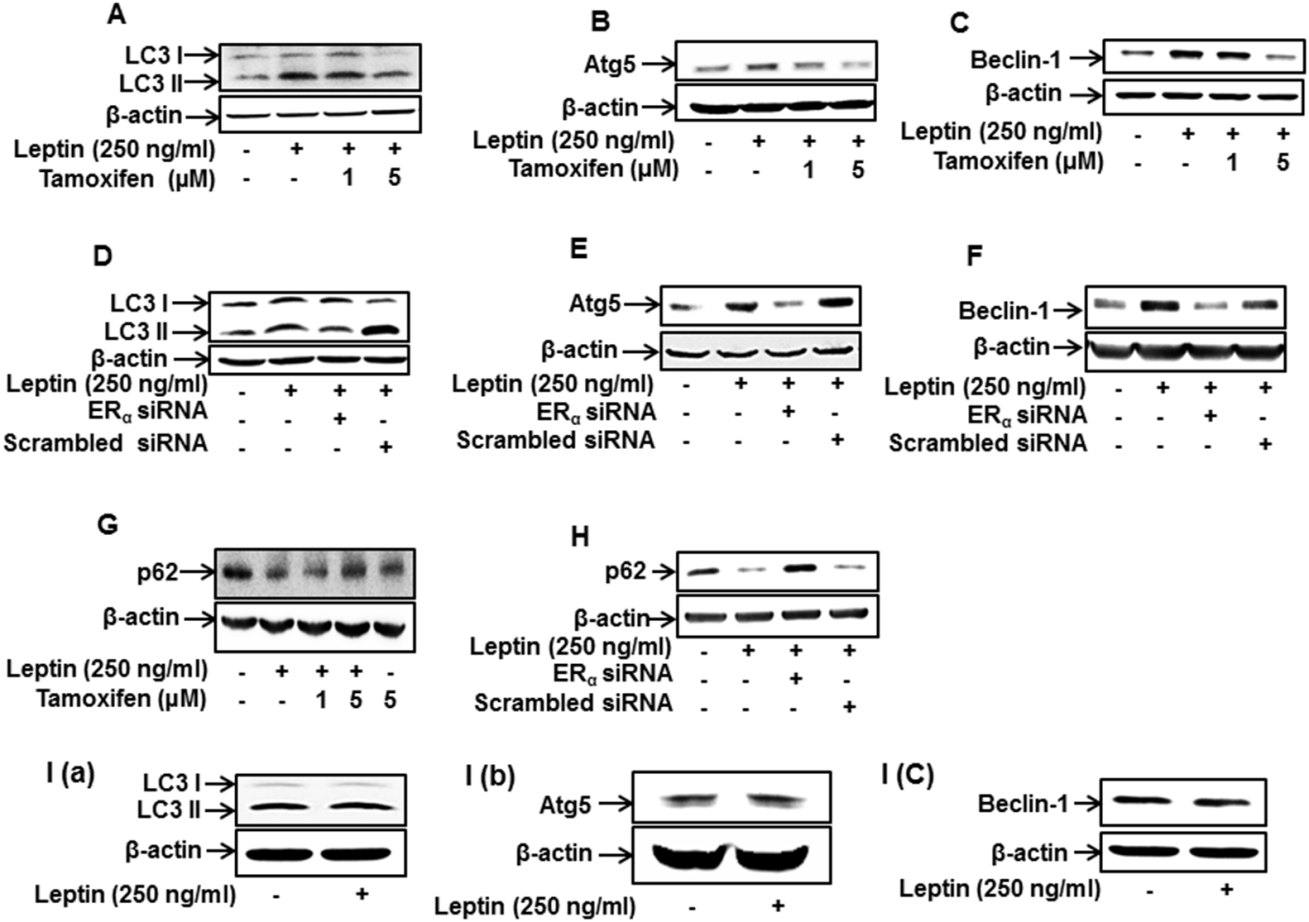

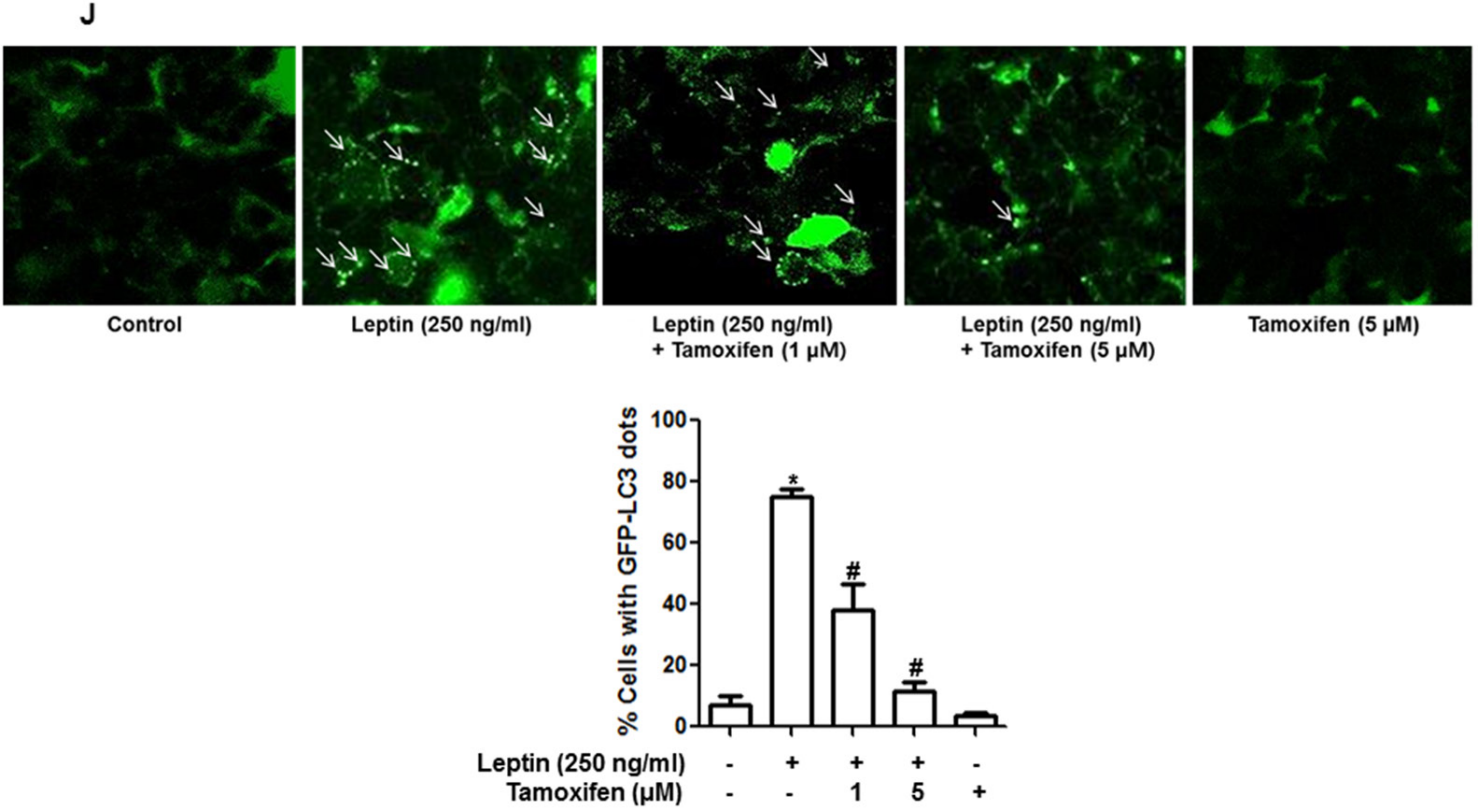
Role of ER signaling in leptin-induced autophagy activation in breast cancer cells **(A-C)** MCF-7 cells were pretreated with the indicated concentrations of tamoxifen for 1 h and subsequently stimulated with leptin (250 ng/mL) for 48 h. Protein expression levels of LC3 (A), Atg5 (B), and Beclin-1 (C) were measured by Western blot analysis. Representative images from at least three independent experiments are shown. **(D-F)** MCF-7 cells were transfected with siRNA targeting ERα. After 48 h of incubation, cells were treated with leptin (250 ng/mL) for 48 h. Protein expression levels of LC3 (D), Atg5 (E), and Beclin-1 (F) were measured by Western blot analysis. Representative images from three independent experiments that showed similar results are shown. **(G** and **H)** MCF-7 cells were pretreated with indicated concentrations of tamoxifen for 1 h (G) or transfected with siRNA targeting the ERα (H) as described previously. Cells were then further stimulated with leptin (250 ng/mL) for 48 h. p62 protein expression was measured by Western blot analysis. **(I)** MDA-MB-231 cells were incubated with leptin (250 ng/mL) for 48 h, and protein expression levels of LC3, Atg5, and Beclin-1 were determined by Western blot analysis. Representative images from three independent experiments are shown. **(J)** MCF-7 cells were transfected with the plasmids expressing eGFP-LC3. After 36 h of incubation, cells were treated with tamoxifen for 1 h and further stimulated with leptin (250 ng/mL) for 48 h. LC3 puncta, representing autophagosome formation, are indicated by white arrows. Images were captured using an A1 confocal laser microscope. Representative images from three independent experiments are shown along with the quantitative analysis data. Values are represented as percentage of the cells expressing GFP-LC3. ^*^ denotes P < 0.05 compared with control cells and ^#^ denotes P < 0.05 compared with the cells treated with leptin but not treated with tamoxifen. Values are presented as mean ± SEM (n=3).

Considering that autophagy induction plays a critical role in leptin-induced tumor growth [11], we next investigated the role of ER signaling in leptin-induced autophagy activation in breast cancer cells. Leptin-induced accumulation of the genes related with autophagy, including LC3II, Atg5, and Beclin-1, was significantly inhibited by pretreatment with tamoxifen (Figure 2A-2C). Essentially similar results were obtained upon transfection with siRNA targeting ERα (Figure 2D-2F). p62, also called sequestosome 1 (SQSTM1), is a marker of autophagic flux. Since p62 is an autophagy cargo protein and is degraded by autophagy induction, p62 cellular levels are inversely correlated with autophagy induction [41]. We also observed that pretreatment with tamoxifen (Figure 2G) and gene silencing of ERα (Figure 2H) restored leptin-induced decrease in p62 expression in MCF-7 cells. Interestingly, leptin did not significantly affect expression of LC3II, Atg5, and Beclin-1 in MDA-MB231 cells (Figure 2I), consistently demonstrating the role of ER signaling in leptin-induced autophagy activation. Finally, we examined the role of ER signaling in autophagosome formation. As expected, pretreatment with tamoxifen significantly suppressed leptin-induced increase in LC3 puncta formation in MCF-7 cells (Figure 2J). Collectively, these results indicate that ER signaling plays a critical role in autophagy induction by leptin in breast cancer cells.

### ER signaling mediates leptin-induced activation of the AMPK/FoxO3A axis in breast cancer cells

**Figure 3:**
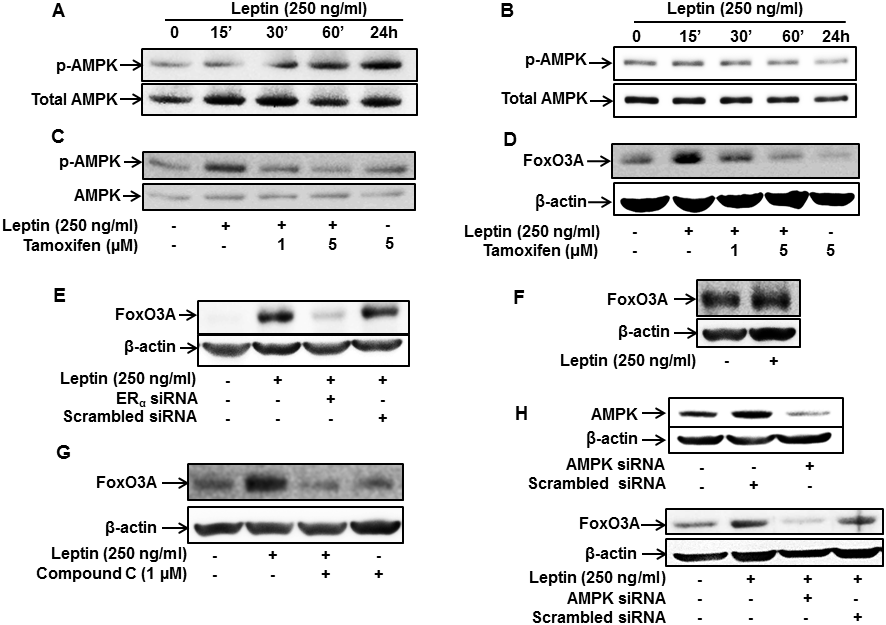
Role of ER signaling in leptin-induced AMPK phosphorylation and FoxO3A expression in MCF-7 cells **(A and B)** MCF-7 cells (A) and MDA-MB-231(B) cells were treated with leptin (250 ng/mL) for the indicated time periods. AMPK phosphorylation was determined via Western blot analysis. Representative images from three independent experiments are shown. **(C)** MCF-7 cells were pretreated with the indicated concentrations of tamoxifen for 1 h, followed by leptin (250 ng/mL) treatment for 24 h. AMPK phosphorylation was measured by Western blot analysis. Representative images from three independent experiments are shown. **(D and E)** MCF-7 cells were pretreated with the indicated concentrations of tamoxifen for 1 h (D) or transfected with 50 nM of siRNA targeting ERα for 48 h (E) and subsequently stimulated with leptin (250 ng/mL) for 48 h. FoxO3A protein expression levels were determined by Western blot analysis as previously described. Representative images from three independent experiments are shown. **(F)** MDA-MB-231 cells were incubated with leptin (250 ng/mL) for 48 h. FoxO3A protein expression levels were measured by Western blot analysis. Representative images from three independent experiments are shown. **(G)** MCF-7 cells were pretreated with Compound C, a pharmacological inhibitor of AMPK, for 1 h followed by treatment with leptin (250 ng/mL) for 48 h. FoxO3A protein levels were determined by Western blot analysis. Representative images from three independent experiments are shown. **(H)** MCF-7 cells were transfected with siRNA targeting AMPKα or control scrambled siRNA (50 nM). (Upper panel) Gene silencing efficiency of AMPKα was assessed by Western blot analysis. (Lower panel) FoxO3A protein levels were examined by Western blot analysis as previously described. Representative images from three independent experiments are shown.

The AMPK/FoxO3A axis plays a critical role in leptin-induced autophagy activation [11, 42]. To further elucidate the molecular mechanisms underlying ER signaling-induced autophagy activation, we examined if ER signaling modulates AMPK/FoxO3A axis. For this, we first assessed the effects of leptin on AMPK phosphorylation in MCF-7 and MDA-MB-231 breast cancer cells. Consistent with the previous reports, leptin treatment rapidly increased AMPK phosphorylation in MCF-7 cells (Figure 3A), whereas no significant effect was observed in MDA-MB-231 cells (Figure 3B). In addition, leptin-induced AMPK phosphorylation was significantly inhibited by tamoxifen pretreatment (Figure 3C), suggesting that leptin induces AMPK activation via an ER-dependent mechanism. We further investigated the role of ER signaling in leptin-induced FoxO3A expression. As shown in Figure 3, pretreatment with tamoxifen or gene silencing of ERα almost completely suppressed FoxO3A induction by leptin in MCF-7 cells (Figure 3D and 3E). Moreover, leptin did not significantly affect FoxO3A expression in MDA-MB-231 cells (Figure 3F), collectively indicating that ER signaling is involved in leptin-induced FoxO3A expression. Finally, inhibition of AMPK signaling via pretreatment with Compound C, a pharmacological inhibitor of AMPK, or gene silencing of AMPK abrogated leptin-induced FoxO3A expression (Figure 3G and 3H), suggesting that AMPK signaling is implicated in FoxO3A induction by leptin. Taken together, these results imply that ER signaling plays a critical role in leptin-induced activation of the AMPK/FoxO3A axis in MCF-7 breast cancer cells.

### ER signaling mediates leptin-induced cell cycle progression via autophagy induction in breast cancer cells

**Figure 4:**
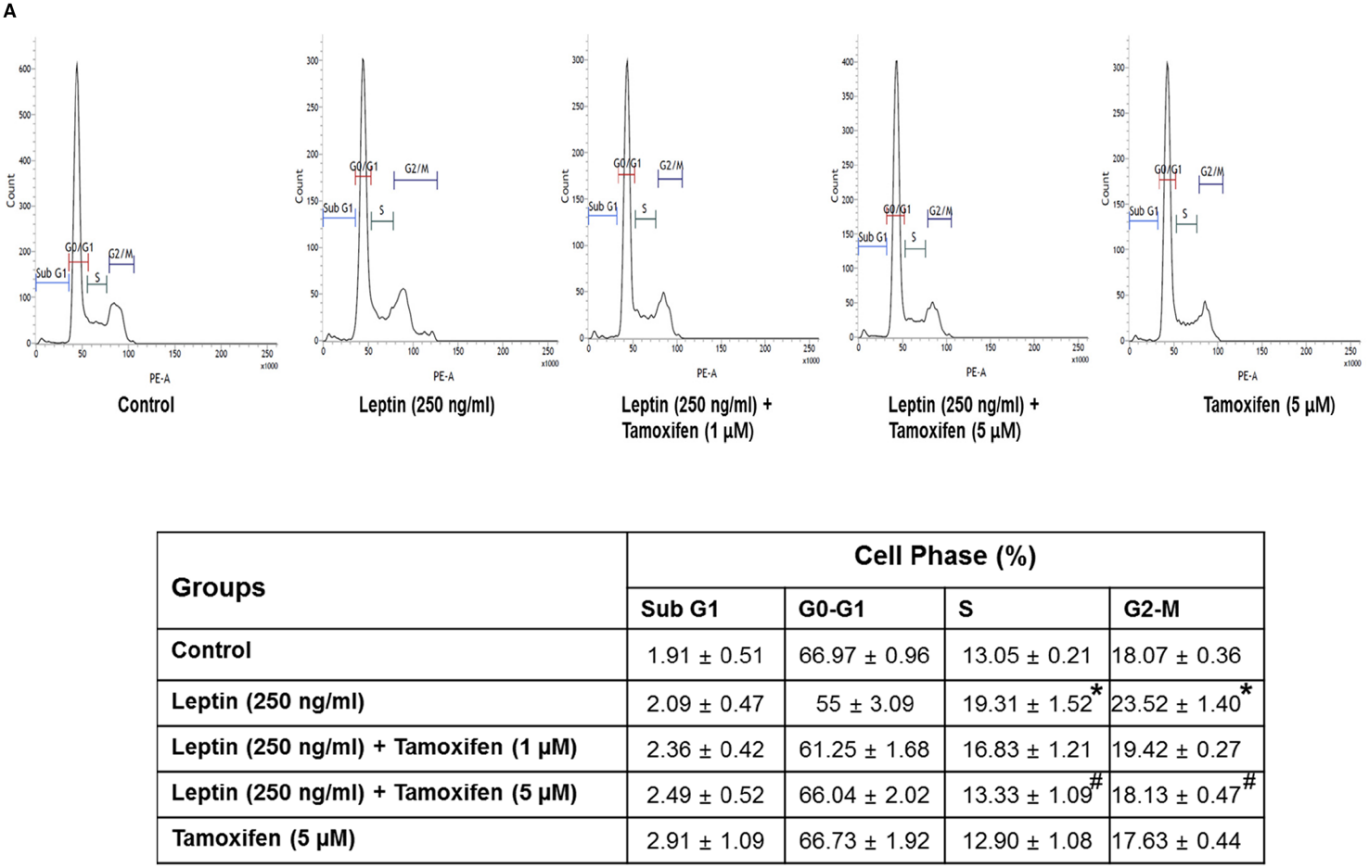

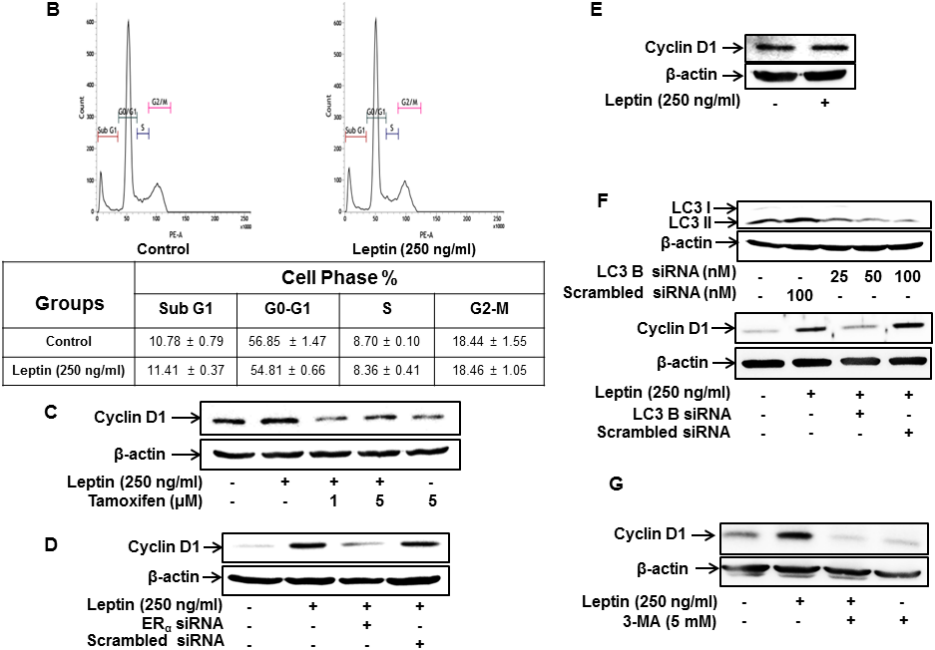
ER signaling and autophagy induction regulate leptin-induced cell cycle progression in breast cancer cells **(A)** MCF-7 cells were pretreated with the indicated concentrations of tamoxifen for 1 h followed by incubation with leptin for 48 h. Percentage of the cells in each cell cycle phase was analyzed using fluorescence activated cell sorting (FACS). Average values are obtained from three independent experiments and presented as mean ± SEM (n=3). ^*^ P < 0.05 compared with control cells. ^#^ P < 0.05 compared with cells treated with leptin but not with tamoxifen. **(B)** MDA-MB 231 cells were treated with leptin (250 ng/mL) for 48 h. Average values are obtained from three independent experiments and presented as mean ± SEM (n=3). **(C and D)** MCF-7 cells were pretreated with indicated concentrations of tamoxifen for 1 h (C) or transfected with siRNA targeting ERα for 48 h (D). Cells were further stimulated with leptin (250 ng/mL) for 48 h. Cyclin D1 protein expression was determined via Western blot analysis as previously described. Representative images from three independent experiments are shown. **(E)** MDA-MB-231 cells were treated with leptin for 48 h. Cyclin D1 protein expression was determined via western blot analysis. Representative images from three independent experiments are shown. **(F)** MCF-7 cells were transfected with siRNA targeting LC3B or control scrambled siRNA for 48 h. (Upper Panel) Gene silencing of LC3B was monitored by Western blot analysis as previously described. (Lower panel) Following transfection, MCF-7 cells were treated with leptin for 48 h. Cyclin D1 expression was measured by Western blot analysis. Representative images from three independent experiments are shown. **(G)** MCF-7 cells were pretreated with 3-methyl adenine (3-MA) for 2 h, followed by leptin treatment for 48 h. Cyclin D1 protein levels were finally determined by Western blot analysis as previously described. Representative images from three independent experiments are shown.

Leptin is known to accelerate cell cycle progression in cancer cells. To elucidate the mechanisms how ER signaling mediates leptin-induced growth of breast cancer cells, we examined the role of ER signaling in leptin-induced cell cycle progression. As shown in Figure 4A, pretreatment with tamoxifen significantly alleviated leptin-induced increase in the populations of the cells in the S and G2-M phases in MCF-7 cells, whereas no significant change in each phase of the cell cycle was observed in MDA-MB-231 breast cancer cells (Figure 4B). To further identify the target molecules during cell cycle modulation, we examined if ER signaling is implicated in leptin-induced expression of cyclin D1, a key cell cycle regulator involved in the G1/S transition phase. Pretreatment with tamoxifen or gene ablation of ERα substantially reduced leptin-induced cyclin D1 expression (Figure 4C and 4D, respectively). Moreover, leptin treatment did not significantly affect cyclin D1 expression in MDA-MB-231 cells (Figure 4E), indicating that leptin-induced cyclin D1 expression is critically regulated via ER signaling. Leptin has been shown to induce the growth of breast cancer cells via autophagy induction. Herein, we further examined the functional role of autophagy in leptin-induced cyclin D1 expression. As shown in Figure 4, gene silencing of LC3B almost completely blocked leptin-induced cyclin D1 expression (Figure 4F). These results were confirmed by treatment with 3-methyladenine (3-MA) (Figure 4G), a pharmacological inhibitor of type III PI3K and used as an autophagy inhibitor. Taken together, these results suggest that ER signaling mediates leptin-induced cyclin D1 expression via autophagy induction.

### Autophagy activation induces cyclin D1 expression through modulating E2F1 expression in MCF-7 cells

**Figure 5:**
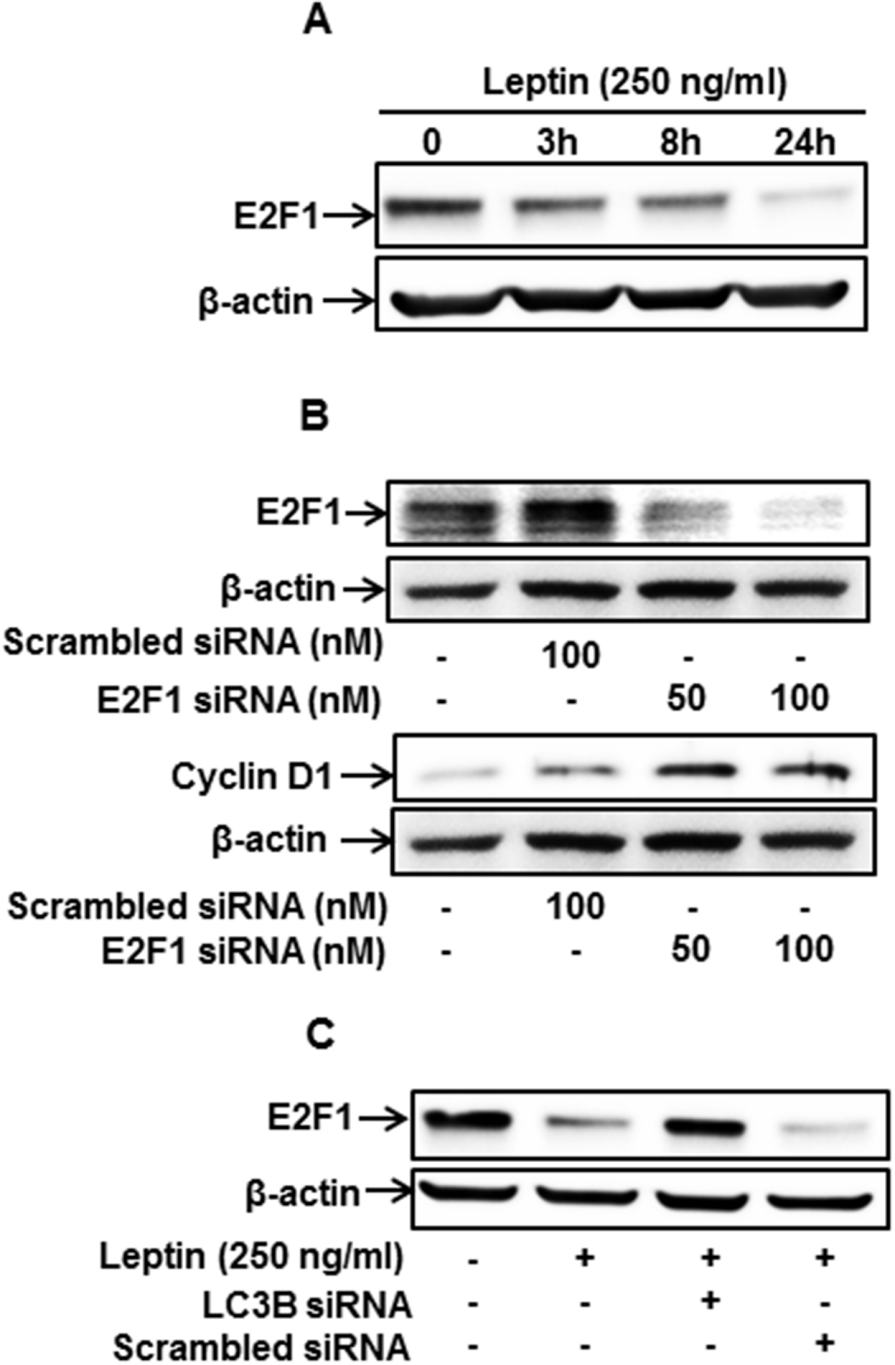
Autophagy induction regulates leptin-induced cyclin D1 expression via modulation of E2F1 in MCF-7 cells **(A)** MCF-7 cells were treated with leptin for the indicated time periods. E2F1 protein levels were determined by Western blot analysis. Representative images from three independent experiments are shown. **(B)** MCF-7 cells were transfected with siRNA targeting E2F1 or control scrambled siRNA for 48 h. (Upper Panel) Gene silencing efficiency of E2F1 was monitored by Western blot analysis. (Lower Panel) Cyclin D1 expression was measured by Western blot analysis. Representative images from three independent experiments are shown. **(C)** MCF-7 cells were transfected with siRNA targeting LC3B for 48 h, followed by leptin treatment for 48 h. E2F1 protein expression was determined by Western blot analysis. Representative images from three independent experiments are shown.

To further identify the upstream molecules involved in autophagy-mediated modulation of cyclin D1 expression, we examined the involvement of E2F1, which is known as a transcriptional repressor for cyclin D1 expression [43]. We first investigated the effect of leptin on E2F1 expression and found that leptin treatment caused a significant decrease in E2F1 protein levels in a time-dependent manner (Figure 5A). In addition, gene silencing of E2F1 led to a significant increase in cyclin D1 expression in MCF-7 cells (Figure 5B), implying that E2F1 acts as a transcriptional repressor of cyclin D1 in our experimental conditions. Finally, interestingly, gene silencing of LC3B almost completely restored the leptin-induced inhibition of E2F1 expression (Figure 5C), collectively indicating that autophagy induction plays a crucial role in leptin-induced E2F1 suppression. Since E2F1 acts as a transcriptional repressor of cyclin D1, suppression of E2F1 level by autophagy would lead to cyclin D1 up-regulation.

### ER signaling is involved in leptin-induced suppression of apoptosis via autophagy induction in MCF-7 breast cancer cells

**Figure 6:**
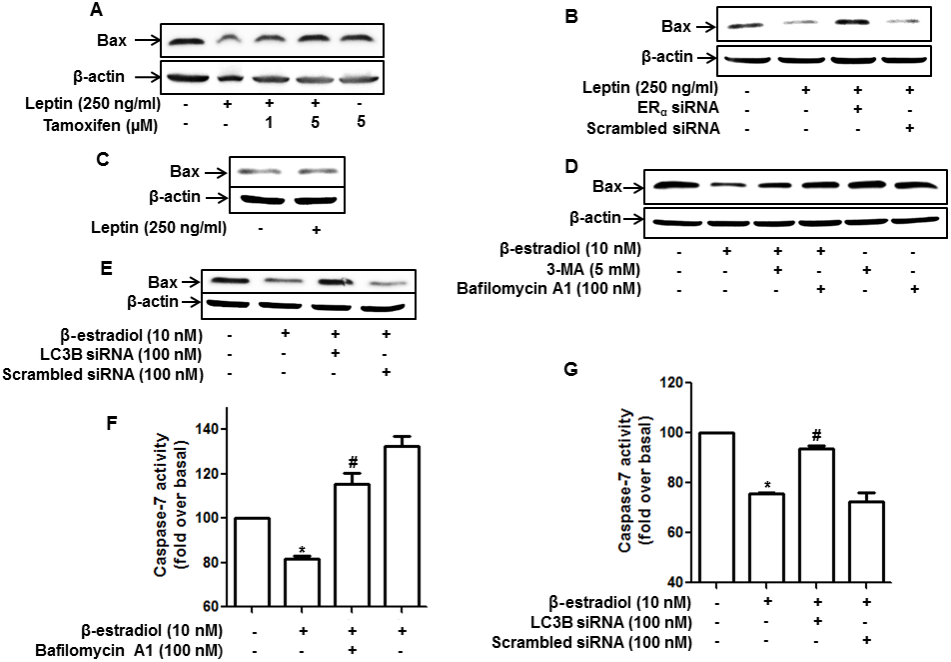
Role of ER signaling and autophagy induction in the regulation of apoptosis and Bax expression in MCF-7 cells **(A and B)** MCF-7 cells were pretreated with the indicated concentrations of tamoxifen for 1 h (A) or transfected with ERα siRNA for 48 h (B) and subsequently treated with leptin for 48 h. Bax protein levels were measured by Western blot analysis. Representative images from three independent experiments that showed similar results are presented. **(C)** MDA-MB-231 cells were treated with leptin for 48 h. Bax protein expression was analyzed via Western blotting. **(D)** MCF-7 cells were pretreated with 3-MA for 2 h or Bafilomycin A1 for 2 h, followed by stimulation with β-estradiol (10 nM) for 24 h. Bax protein levels were determined by Western blot analysis. Representative images from three independent experiments are shown. **(E)** MCF-7 cells were transfected with siRNA targeting LC3B for 48 h and further stimulated with β-estradiol for 24 h. Bax protein levels were measured by Western blot analysis. Representative images from three independent experiments are shown. **(F and G)** MCF-7 cells were pretreated with bafilomycin A1 for 2 h (F) or transfected with siRNA targeting LC3B for 48 h (G), followed by stimulation with β-estradiol for 48 h. Caspase-7 activity was determined as described in the Materials and Methods section. Values are presented as mean ±SEM (n=3). ^*^ denotes P < 0.05 compared with control cells. ^#^ denotes P < 0.05 compared with cells treated with β-estradiol but not pretreated with bafilomycin or transfected with LC3B siRNA.

Growth of cancer cells is determined by the modulation of apoptosis, as well as the cell cycle. Leptin treatment has been reported to suppress apoptosis and Bax expression via autophagy induction [11]. In the present study, we investigated the role of ER signaling in leptin-induced suppression of Bax expression in MCF-7 cells. As shown in Figure 6, suppression of Bax expression was substantially restored by pretreatment with tamoxifen (Figure 6A). Gene silencing of ERα also significantly abrogated Bax suppression in MCF-7 cells (Figure 6B), whereas leptin did not significantly affect Bax expression in MDA-MB-231 cells (Figure 6C). Taken together, these results suggest that ER signaling plays an important role in leptin-induced suppression of Bax expression probably via autophagy induction in breast cancer cells. To confirm the role of ER signaling and autophagy induction in the modulation of Bax expression and subsequent apoptosis, we examined the effect of β-estradiol on Bax expression and the role of autophagy induction in this process. As shown in Figure 6D, consistent with previous reports, β-estradiol treatment significantly reduced Bax expression. In addition, Bax inhibition was restored by pretreatment with 3-MA or bafilomycin, a selective inhibitor of vacuolar H^+^ ATPase. Moreover, gene silencing of LC3B showed essentially similar results (Figure 6E), demonstrating the pivotal role of autophagy induction in the suppression of Bax expression by ER signaling. Furthermore, inhibition of autophagy by treatment with bafilomycin A1 (Figure 6F) or knockdown of LC3B (Figure 6G) also significantly restored suppression of caspase-7 activity by β-estradiol in MCF-7 cells, thereby confirming that ER signaling negatively regulates apoptosis in breast cancer cells via autophagy induction.

### ER signaling mediates leptin-induced tumor growth via autophagy induction in MCF-7 tumor xenograft model

**Figure 7:**
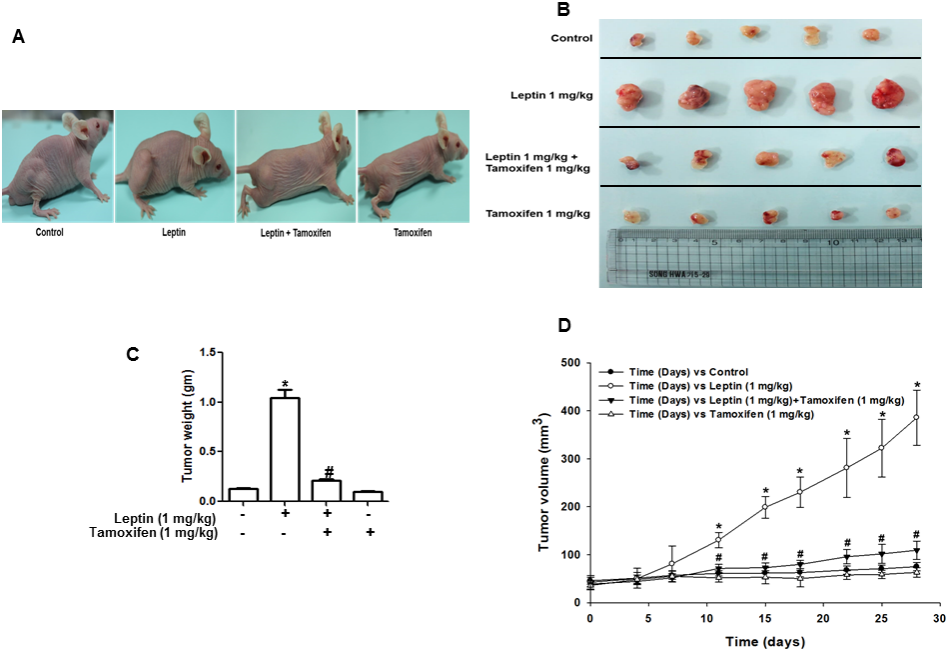

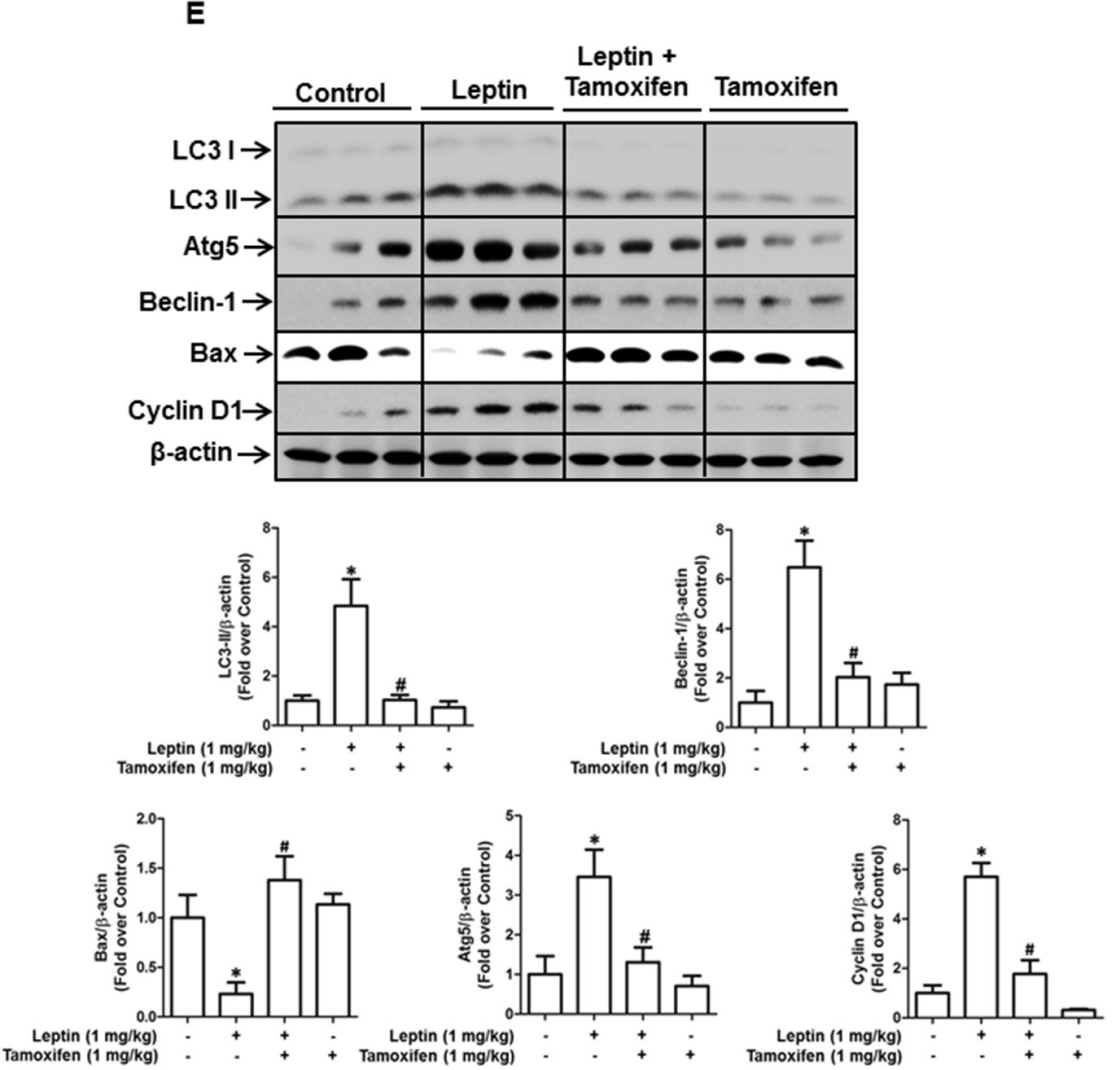
Role of ER signaling in leptin-induced growth of MCF-7 tumor xenograft model MCF-7 tumor xenograft model was established using 4-week-old BALB/c nude male mice. MCF-7 cells were injected subcutaneously into the rear flank of the mice. After 10 days of subcutaneous injection of MCF-7 cells, mice were randomly divided into the following four groups: control, leptin (1 mg/kg), leptin (1 mg/kg) and tamoxifen (1 mg/kg), and tamoxifen (1 mg/kg) alone. Leptin and tamoxifen were intraperitoneally administered every 36 h and 24 h, respectively, for 4 weeks. **(A)** Representative images of mice from each group at the end of the treatment. **(B)** After four weeks of treatment, tumor tissues were collected and represented. **(C)** Tumor tissues were collected, and the corresponding weights were measured. Values are presented as mean ± SEM (n=5). ^*^ P < 0.05 compared to the control mice. ^#^ P<0.05 compared to the mice treated with leptin. **(D)** During treatment, tumor volume was measured twice weekly as described in the Materials and Methods section. **(E)** Tumor tissues were lysed as indicated in the Materials and Methods section, and protein expression levels of autophagy-related genes, including LC3, Atg5, and Beclin-1, a cell cycle-related gene (cyclin D1), and an apoptotic gene (Bax) were determined in different treatment groups by Western blot analysis. Quantitative analyses of protein expression of LC3, Atg5, Beclin-1, cyclin D1 and Bax were determined by densitometric analysis and shown in the lower panel. Values are presented as mean ± SEM (n=5). ^*^ P < 0.05 compared to the control mice. ^#^ P<0.05 compared to the mice treated with leptin.

Based on the results obtained from *in vitro* studies using ER-positive and ER–negative breast cancer cells, we demonstrated that ER signaling mediates leptin-induced growth of breast cancer cells via autophagy induction. To validate the results obtained from *in vitro* experiments, we prepared MCF-7 tumor xenografts in BALB/c nude mice and examined the role of ER signaling in leptin-induced autophagy induction and tumor growth. As expected, leptin administration accelerated the growth of MCF-7 cells in a xenograft model (Figure 7A). The tumor growth-promoting effects of leptin were also confirmed by measuring tumor size (Figure 7B), tumor weight (Figure 7C), and tumor volume (Figure 7D). Interestingly, co-treatment with tamoxifen prevented leptin-induced tumor growth, indicating that ER signaling is crucial for leptin-induced tumor growth in our *in vivo* experimental conditions. We further examined the functional role of ER signaling in autophagy induction in a xenograft model. As shown in Figure 7E, consistent with the *in vitro* results, tamoxifen treatment significantly suppressed leptin-induced up-regulation of autophagy-related genes, including LC3II, Atg5, and Beclin-1. In addition, leptin-induced suppression of Bax expression was almost completely recovered by co-treatment with tamoxifen (Figure 7E), implying the involvement of ER signaling in the regulation of Bax expression and further apoptosis by leptin, which are also in agreement with the results obtained from *in vitro* studies. Finally, leptin-induced cyclin D1 expression was also significantly decreased upon co-administration with tamoxifen. In conclusion, these results further verify the critical role of ER signaling in leptin-induced autophagy activation and highlight its critical role in the inhibition of apoptosis and cell cycle progression in an *in vivo* model.

## DISCUSSION

A number of epidemiological studies have demonstrated that obesity is closely associated with increased incidence of various types of cancer, especially liver, colon, and breast cancers [44–46]. However, the underlying mechanisms by which obesity contributes to the development and progression of cancer remain largely unknown. One of the plausible mechanisms is through alterations in adipokine levels in obese individuals. In particular, levels of circulating adiponectin are lower in obese patients, whereas leptin levels are significantly higher. Given that adiponectin exerts potent anti-tumor properties but leptin promotes tumor growth [9, 47], changes in adiponectin and leptin levels could be attributed to high incidence of cancer in obese patients. Indeed, increased blood leptin levels have been clearly demonstrated to play a role in the development and progression of various types of cancers. However, the detailed molecular mechanisms are still largely unknown. In the present study, we attempted to elucidate the underlying mechanisms, with focus on the role of ER signaling in relation to autophagy induction. Herein, we demonstrated that ER signaling plays a crucial role in both leptin-induced cell cycle progression and apoptosis inhibition through autophagy induction in breast cancer cells.

ER signaling is well known to be associated with the pathogenesis of breast cancer by demonstrating that excess production of estrogens due to enhanced aromatase activity or higher conversion rates of androgens to estrogens increases the risk for the development of breast and endometrium cancer [48]. Moreover, activation of estrogen signaling promotes tumor formation in breast cancer cells [49, 50]. A number of hypotheses have been proposed as mechanisms underlying estrogen-induced tumor growth. For example, ER activation leads to an increase in ROS production, which plays a pivotal role in cell cycle progression and migration of the breast cancer cells [51]. In addition, estrogen levels are positively correlated with IL-1β expression in breast tissue and that estrogen stimulates IL-1β production in breast cancer xenografts [52, 53], raising a possibility that the inflammasome activation could be involved in estrogen-induced tumor growth. Although it is well known that ER signaling leads to tumor progression, the detailed molecular mechanisms are not clearly understood. In this study, we clearly demonstrated that ER signaling plays a crucial role in leptin-induced autophagy activation, which in turn facilitates the growth of breast cancer cells via both apoptosis inhibition and cell cycle progression.

As described earlier, estrogen is known to induce the growth of breast cancer cells primarily by activating ERα. When estrogen binds to its receptor, it undergoes conformational changes and translocates to the nucleus, where it binds to the promoter regions of target genes and initiates transcriptional activation. In addition to the ligand binding-induced activation, the ER can also be activated in the absence of estrogen, a process referred to as transactivation. Leptin has been demonstrated to activate the ER by stimulating MAPK signaling [29, 30]. In addition, leptin prevents ubiquitination and degradation of ER induced by ICI-182, 780, an anti-estrogenic modulator, in breast cancer cells and thereby develops resistance to anti-estrogen therapy [13]. In the present study, we demonstrated that leptin promotes the growth of ER-positive breast cancer cells but does not significantly affect ER-negative breast cancer cells (Figure 1A and 1B), suggesting that ER signaling is involved in leptin-induced growth of breast cancer cells. In continuing experiments to investigate the role of ER signaling in autophagy induction, we observed that leptin increased the expression of autophagy-related genes (ATGs) in MCF-7 cells without significant effect on ER-negative cells (Figure 2). Furthermore, leptin-induced increases in up-regulation of ATGs and autophagosome formation were significantly suppressed by pretreatment with a pharmacological inhibitor of ER or genetic ablation of ERα (Figure 2A-2F and 2J). Thus, the results from the current study together with the previous reports demonstrate that ER signaling plays a crucial role in leptin-induced growth of breast cancer cells most probably through autophagy activation. In contrast to the results observed in the current study, a recent study reported that treatment with estrogen or estrogen agonist inhibited leptin-induced growth of hepatic cancer cells by facilitating apoptosis [50]. In this study, we also found that tamoxifen treatment did not significantly affect leptin-induced growth of hepatic cancer cells (Supplementary Figure 1), indicating that estrogen receptor does not mediate leptin-induced growth of hepatic cancer cells. The detailed mechanisms underlying differential roles of estrogen receptor signaling in leptin-induced tumor growth between MCF-7 breast cancer cells and HepG2 hepatic cancer cells remains to be delineated. One of the plausible mechanisms is that each estrogen receptor isoform (ER-α, ER-β, and G protein-coupled estrogen receptor) exhibits different roles in tumor growth and, therefore, it is possible that differential roles of estrogen receptor signaling could be due to different formation (proportion) of the estrogen receptor isoforms between hepatic and breast cancer cells. Future studies to identify the specific estrogen receptor isoform involved in the modulation of tumor growth would provide comprehensive understanding of the role of estrogen receptor signaling in modulating the growth of hepatic and breast cancer.

While various mechanisms have been proposed as the mechanisms underlying ER signaling-induced tumor growth, the involvement of autophagy induction has not been reported, although autophagy is well known to modulate cancer growth. As described earlier, autophagy exerts its biological roles by eliminating intracellular components through the autolysosome machinery. For example, autophagy negatively regulates apoptosis via targeting and degradation of Bax [54]. Leptin suppressed apoptosis in cancer cells via autophagy induction and degradation of Bax, as mentioned earlier. In addition to apoptosis inhibition, accumulated evidence indicate that leptin promotes cell cycle progression in ovarian cancer cells and breast cancer cells via enhancing expression of the genes involved in cell cycle progression [12, 55] but suppress the expression of cell cycle negative regulators, including p21 and p27 [55]. In the present study, we further investigated the role of ER signaling and autophagy induction in leptin-induced cell cycle progression. Herein, we showed that genetic or pharmacological ablation of ER signaling suppressed leptin-induced cyclin D1 expression in MCF-7 cells (Figure 4C and 4D). Moreover, blockade of autophagy induction also inhibited leptin-induced cyclin D1 expression (Figure 4F and 4G), indicating that cyclin D1 would be a target molecule for autophagy- and ER signaling-mediated suppression of cell cycle progression by leptin in breast cancer cells. To further elucidate the role of ER signaling in the modulation of the cell cycle regulators, we have examined the effects of leptin and tamoxifen on the expression of cyclin E, p27, and p21. We observed that leptin substantially increased expression of cyclin E, but suppressed p27, while leptin did not significantly suppressed p21 in this experimental condition (data not shown). In addition, tamoxifen suppressed leptin-induced increase in cyclin E, and restored suppression of p27 (Supplementary Figures 2 and 3). These results raise a possibility that p27 and cyclin E could be another candidate molecules involved in the modulation of cell cycle by leptin and estrogen receptor signaling, but p21 would not be implicated in the progression of cell cycle by leptin and ER signaling.

The E2 factor (E2F) family of transcription factors comprises critical regulators of the cell cycle. In particular, E2F1 is known as a transcriptional repressor of cyclin D1, as indicated that E2F1 overexpression leads to significant inhibition of cyclin D1 expression [43]. To further elucidate the mechanisms underlying autophagy-induced increase in cyclin D1 expression, we examined whether autophagy induction modulates E2F1 expression. In this study, we showed that leptin treatment decreased E2F1 protein expression in MCF-7 cells (Figure 5A), consistent with the previously reported microarray analysis [56]. We further demonstrated that gene silencing of E2F1 leads to cyclin D1 up-regulation and that gene silencing of LC3B restores leptin-induced suppression of E2F1 expression (Figure 5B and 5C). Taken together, these findings clearly demonstrate that autophagy targets and degrades E2F1, thereby leading to cyclin D1 induction in leptin-treated MCF-7 breast cancer cells. In fact, the role of E2F1 in cancer modulation is controversial and it acts as both a tumor promoter and suppressor. For example, overexpression of E2F1 in a liver disease model of transgenic mice caused dysplasia and tumor formation [57]. Likewise, E2F1 overexpression led to activation of the Ras oncogene and promoted skin tumor development [58]. Conversely, E2F1 overexpression in transgenic mice suppressed hair growth due to apoptosis induction in the hair follicles [58]. Moreover, loss of E2F1 resulted in the development of reproductive tract sarcoma, lung cancer, and lymphomas in mice [59], collectively indicating a tumor suppressive role for E2F1. Herein, we demonstrated that E2F1 suppression by autophagy induction is closely associated with leptin-induced cell cycle progression in breast cancer cells. To the best of our knowledge, this is the first report demonstrating the role of autophagy activation in modulating E2F1 and cyclin D1 expression.

Both AMPK and FoxO3A are key regulators of cellular metabolism, and the AMPK/FoxO3A axis was originally reported as an energy sensor pathway [38, 60]. In addition to its important role in maintaining the homeostasis of energy and metabolism, recent studies indicate that the AMPK/FoxO3A pathway is activated under conditions of metabolic stress and plays a role in determining the fate of the cells into either cell death or cell survival [61]. The AMPK/FoxO3A axis has been recently shown to trigger autophagic cell death or induce cancer cell survival depending on cellular context [62]. FoxO3A has been shown to exert a prominent role as a transcriptional activator responsible for induction of the genes related with autophagy [11]. The transcriptional activity of FoxO3A is modulated by subcellular localization and/or phosphorylation status, which is modulated by various types of kinases. For example, phosphorylation of FoxO3A by AKT leads to export from nucleus to the cytosol causing suppression of the target genes, while phosphorylation by JNK promotes nuclear localization leading to the enhancement of the transcription [63]. In addition to the modulation by subcellular localization, FoxO3A protein expression is modulated via multiple mechanisms, including regulation at transcription level, protein stability, and degradation [64]. In the present study, we found that leptin treatment induced increase in FoxO3A protein expression. Moreover, inhibition of AMPK signaling inhibited leptin-induced FoxO3A expression (Figure 3G and 3H), clearly demonstrating that leptin-induced FoxO3A expression is mediated by AMPK signaling-dependent mechanism in MCF-7 breast cancer cells.

Herein, we have also shown that leptin induces AMPK phosphorylation in breast cancer cells through ER-dependent mechanism (Figure 3A and 3C). AMPK activity can be regulated via various mechanisms depending on experimental condition. For instance, AMPK is phosphorylated and activated by upstream kinase. To date, Liver kinase B1 (LKB1) and Calmodulin-dependent protein kinase kinase (CAMKK2) have been identified as upstream kinases, which phosphorylate AMPK. In addition, a recent study reported that ER caused AMPK activation via direct binding with catalytic-α-subunit of AMPK. Furthermore, ER can interact with LKB1 complex, an upstream of AMPK, which in turn leads to activation of AMPK [65]. In this study, we could not thoroughly address how estrogen receptor signaling leads to AMPK phosphorylation in our experimental condition. Further studies will be needed to gain more insights into the mechanisms by which leptin and estrogen receptor induces activation of AMPK signaling in cancer cells.

In the present study, we found that the AMPK/FoxO3A axis is involved in leptin-induced autophagy activation and contributes to the growth of cancer cells. Furthermore, we showed that ER inhibition abolished leptin-induced AMPK phosphorylation (Figure 3A and 3C) and FoxO3A expression in breast cancer cells (Figure 3E and 3F). Therefore, our results clearly demonstrate the role of ER signaling in the activation of AMPK/FoxO3A axis by leptin in breast cancer cells. In contrast to these findings, Zekas and colleagues recently reported that activation of estrogenic signaling resulted in translocation of FoxO3A from nucleus to cytoplasm via G-protein coupled estrogen receptor (GPER)-dependent mechanisms [66], suggesting differential roles of each ER isoform in FoxO3A expression and its activity. Although, herein, we demonstrated the role of ER signaling in the activation of the AMPK/FoxO3A axis, further studies will be required to decipher the molecular mechanisms underlying differential roles of ER isoforms in the regulation of FoxO3A expression/activity.

The AMPK/FoxO3A axis has been demonstrated to be involved in tumor growth by suppressing apoptosis [67]. Herein, we further examined the role of the AMPK/FoxO3A axis in leptin-induced cell cycle progression. The results from the present study revealed that pretreatment with Compound C and genetic ablation of AMPK prevented leptin-induced increase in cyclin D1 expression in MCF-7 cells (Supplementary Figures 4 and 5), indicating that the AMPK/FoxO3A axis could be involved in cell cycle progression. To the best of our knowledge, this is the first report demonstrating the crucial role of the AMPK/FoxO3A axis and autophagy induction in leptin-induced cell cycle progression. Interestingly, in contrast to the results observed in this study, AMPK/FoxO3A axis has been shown to lead to cell cycle arrest induced by adiponectin. Adiponectin, a hormone also derived from adipose tissue, has been demonstrated to inhibit tumor growth [68]. Additionally, adiponectin induces diverse physiological responses that oppose the effects induced by leptin. However, the mechanisms underlying the opposing effects of adiponectin are not clearly understood. Given that the two prototypical adipokines (adiponectin and leptin) induce a number of opposing biological responses, further studies seeking to elucidate the molecular mechanisms underlying the differential role of the AMPK/FoxO3A axis between leptin- and adiponectin-induced modulation of tumor growth would be helpful for identification of promising pharmacological target molecules that could be useful in developing effective strategies for the treatment of cancer.

**Figure 8:**
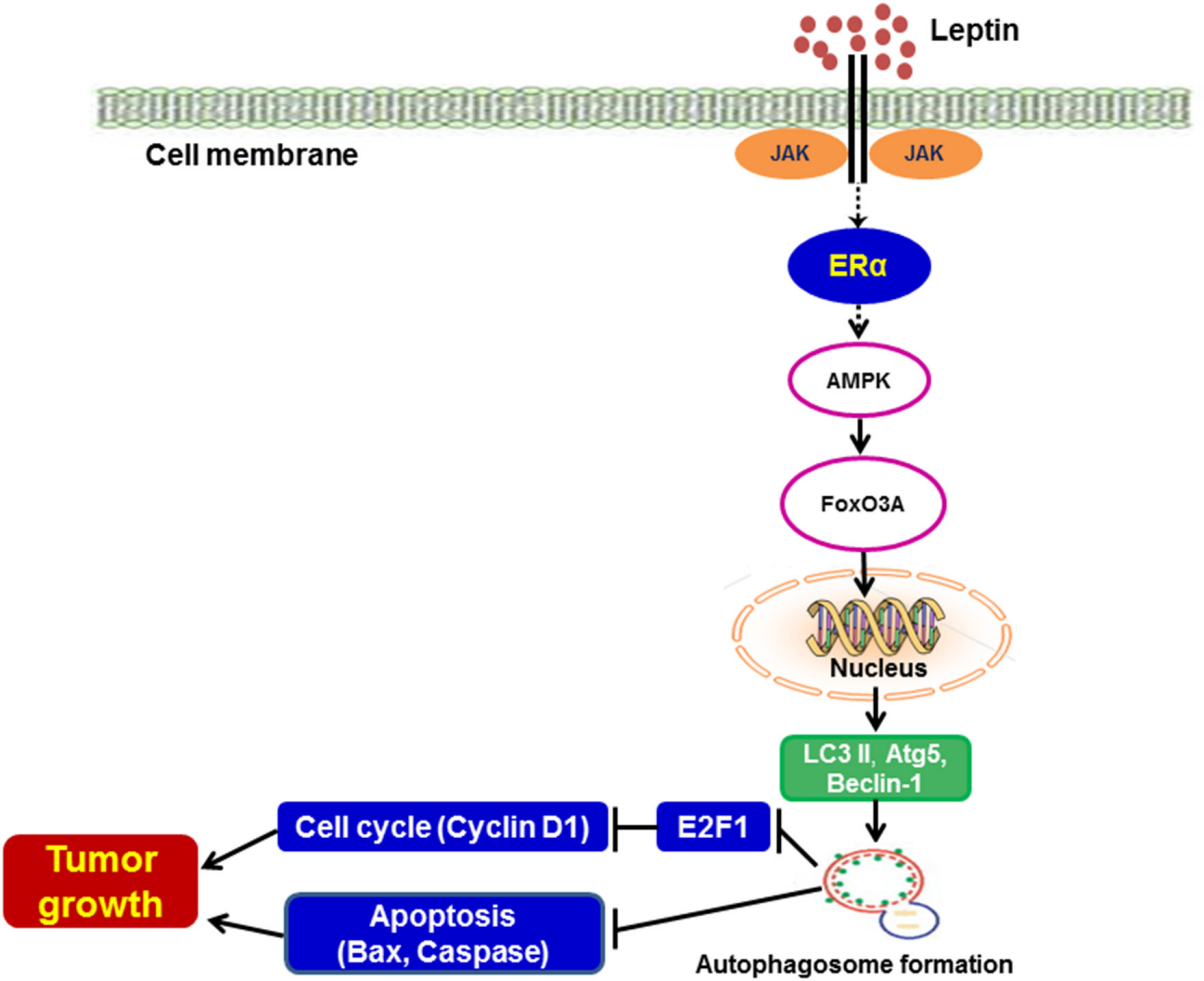
Proposed model for the involvement of estrogen receptor signaling and autophagy activation in leptin-induced growth of breast cancer cells Leptin has been shown to induce the growth of breast cancer cells through various mechanisms. Autophagy induction is known as an important survival mechanism that promotes tumor growth. In the present study, we demonstrated that leptin induced autophagy activation via ER signaling-dependent mechanisms both in MCF-7 breast cancer cells and in a cancer xenograft *in vivo* model. ER signaling-mediated autophagy activation negatively regulates apoptosis via Bax suppression and also promotes cell cycle progression by modulating E2F1 and cyclin D1 expression. In addition, leptin-induced increase in the expression of autophagy-related genes was mediated via AMPK phosphorylation and FoxO3A induction. Further studies are required to identify the specific estrogen receptor type implicated in this process and its mechanism of action. Moreover, future studies are needed to elucidate the molecular mechanisms underlying autophagy activation by AMPK/FoxO3A axis.

In conclusion, the results from both *in vitro* and *in vivo* models clearly demonstrated that ER signaling plays a crucial role in leptin-induced growth of breast cancer cells via autophagy induction. Tumor growth via ER signaling and autophagy induction is mediated via both inhibition of apoptosis and promotion of cell cycle progression. In addition, ER signaling utilizes the AMPK/FoxO3A axis to induce autophagy in leptin-treated breast cancer cells (Figure 8). Results from the present study indicate that suppression of ER signaling and autophagy induction would be a novel strategy that can be used to suppress leptin-induced growth of breast cancer.

## MATERIALS AND METHODS

### Materials

All cell culture reagents were purchased from Hyclone laboratories (South Logan, Utah, USA). Recombinant mouse leptin, tamoxifen, bafilomycin A1, and 3–methyladenine (3-MA) were procured from Sigma-Aldrich (St. Louis, MO, USA). Cell proliferation and Caspase-7 activity assay kits were purchased from Promega Corporation (Madison, WI, USA). Cycletest™ Plus DNA Kit was purchased from BD Biosciences (San Jose, CA, USA). Antibodies targeting LC3, Beclin-1, Bax, FoxO3A, ERα, p27 and AMPKα(total and phospho-specific) were obtained from Cell Signaling Technology, Inc. (Beverly, MA, USA). Antibody against Atg5 was obtained from Thermo Scientific, Inc. (Rockford, IL, USA). Antibodies specific to cyclin E and Cyclin D1 were procured from Santa Cruz Biotechnology (Santa Cruz, CA, USA). All other chemicals were purchased from Sigma-Aldrich unless mentioned otherwise.

### MCF-7 and MDA-MB-231 cell culture

The MCF-7 cell line was purchased from American Type Culture Collection (ATCC, Rockville, MD, USA) and routinely cultured in Dulbecco’s Modified Eagle Medium (DMEM) with 10% (v/v) Fetal Bovine Serum (FBS) and 1% (v/v) Penicillin-streptomycin. For the treatment with β-estradiol, cells were cultured in phenol red-free DMEM with charcoal-stripped 10% (v/v) FBS and 1% (v/v) penicillin-streptomycin. The MDA-MB-231 cell line was purchased from Korean Cell Line Bank (KCLB, Seoul, South Korea) and routinely cultured in RPMI 1640 supplemented with 10% (v/v) fetal bovine serum and 1% (v/v) penicillin-streptomycin. Cells were cultured at 37 °C in an incubator with humidified atmosphere containing 95% oxygen and 5% CO_2_.

### Measurement of cell viability (MTS assay)

Cell viability/growth was assessed as previously described [69]. Briefly, cells were seeded in 96-well plates at a density of 2×10^4^ cells/well. After overnight culture, cells were treated with leptin for the indicated time periods and further incubated with 3-(4,5-dimethylthiazol-2-yl)-5-(3-carboxymethoxyphenyl)-2-(4-sulfopheny)-2H-tetrazolium (MTS) solution for an additional 2 h. Cell viability was determined by measuring the amount of formazan product at an absorbance of 490 nm using a SPECTROstar^Nano^ microplate reader (BMG Labtech Inc., Germany).

### Caspase-7 activity assay

Caspase-7 activity was determined using Caspase-Glo^®^ 3/7 assay kits (Promega Corporation, Madison, USA) according to the manufacturer’s instructions. Briefly, cells were seeded at a density of 2×10^4^ cells/well in 96-well plates. After overnight culture, cells were treated with leptin and other agents as indicated. Caspase-7 activity was determined by measuring the luminescence generated by the cleavage of luminogenic substrate Ac-DEVD-pNA using a microplate reader (Flurostar Optima, BMG Labtech, Germany).

### Transient transfection with small interfering RNA (siRNA)

Cells were seeded at a density of 3×10^5^ cells/35-mm dish. After overnight incubation, cells were transfected with siRNA targeting the genes of interest or scrambled control siRNA using HiPerfect transfection reagent (Qiagen, Maryland, USA) following the manufacturer’s instructions. Gene silencing efficiency was monitored via Western blot analysis after 24 or 48 h of transfection. siRNA duplexes used in this study were obtained from Bioneer (Daejeon, South Korea). Sequences of the siRNA duplexes are listed in Table 1.

**Table 1:** Sequences of small interfering RNA for transfection

Target gene	Primer	Nucleotide sequence
Estrogen Receptor alpha(ERα)	F R	5’- GUCACUACUCAGGCUGACU -3’ 5’- AGUCAGCCUGAGUAGUGAC -3’
LC3B	F R	5’- GACUGUCUCGUUUAGACUG -3’ 5’- CAGUCUAAACGAGACAGUC -3’
AMPKα	F R	5’- CUGAGUUGCAUAUACUGUA -3’ 5’- UACAGUAUAUGCAACUCAG -3’
Scrambled control	F R	5’- CCUACGCCACCAAUUUCGU -3’ 5’- ACGAAAUUGGUGGCGUAGG -3’

### Confocal microscopic analysis

To measure LC3 puncta formation, MCF-7 cells were seeded at a density of 3×10^5^ cells/cover glass-bottom dish. After overnight incubation, cells were transfected with enhanced green fluorescent protein (eGFP)-LC3 expression plasmid using Fugene HD transfection reagent (Promega, Madison, USA) according to the manufacturer’s instructions. After 36 h of plasmid transfection, cells were treated with leptin in the presence or absence of tamoxifen. Cells were then washed with PBS and fixed with 4% paraformaldehyde solution. Confocal images were captured using an A1 Confocal Laser Microscope System (Nikon Corp., Tokyo, Japan). Autophagy puncta formation was quantified as previously described [70] from triplicate experiments. Confocal images were expressed as percentage of cells with GFP-LC3 dots observed from at least 100 cells using Image Inside software version 2.32.

### Cell cycle analysis

Cell cycle analysis was performed using Cell cycle test plus DNA reagent kit (BD Biosciences) following the manufacturer’s instructions. Briefly, cells were seeded in 6-well plates at a density of 2×10^5^cells/well. After overnight culture, cells were treated with leptin and/or tamoxifen for the indicated time duration. After washing with PBS and buffer solution, Buffer solution A, solution B, and the propidium iodide solution were sequentially added to the cells, followed by incubation for 15 min in ice on dark. Finally, DNA contents in the cells stained with propidium iodide were determined using a flow cytometer (BD FACSverse). Cell distribution in different phases of the cell cycle was analyzed using BD FACSuite software.

### Preparation of cellular extracts, tumor lysates and Western blot analysis

To determine the protein expression levels of the target genes, total cellular extracts were isolated using RIPA lysis buffer containing halt protease inhibitor cocktail (Thermo Scientific). Tumor lysates used for Western blot analysis were prepared as previously described [11] with slight modifications. About 100 mg of tumor tissue was homogenized in ice-cold PBS containing 1% protease inhibitors. After centrifugation at 1,500 *g* for 3 min, the supernatants containing blood and debris were removed, while the remaining tissue pellets were further lysed using a Sonics Vibra-Cell ultrasonicator (Sonics and Materials Inc., USA). After centrifugation at 13,000 *g* for 15 min, supernatants were collected and further subjected to Western blot analysis as mentioned previously. For immunoblot analysis, 20–30 μg of total protein was separated using SDS-PAGE (8–15% of SDS-polyacrylamide gel) and transferred to PVDF membranes. Membranes were then blocked with 5% skim milk for 1 h, incubated with the designated primary antibodies (1:1000 dilution in 3% BSA) at 4°C overnight, and further incubated with horseradish peroxidase-conjugated secondary antibodies (1:4000 in 3% BSA). Finally, chemiluminescent images were captured using a Fujifilm LAS-4000 mini (Fujifilm, Tokyo, Japan). Membranes were then stripped and reprobed with antibodies against β-actin, which was used as a loading control.

### Establishment of MCF-7 tumor xenografts

All animal experiments were conducted following the guidelines approved by the Yeungnam University Research Committee for the Care and Use of Laboratory animals. For the development of tumor xenografts, MCF-7 cells (1×10^7^ cells/200 μl) were injected into the rear flanks of four-week-old male BALB/c nude mice procured from Orient Ltd. (Osan, South Korea). After 10 days of implantation, mice were randomly divided into four groups (n=5 per group). Leptin was injected intraperitoneally every 36 h, and tamoxifen was administered every 24 h for the whole duration of the treatment. Tumor size was measured twice a week using a digital Vernier caliper and calculated using the following equation: V= (width)^2^ × length/2. After four weeks of treatment, mice were sacrificed, and tumors were excised, weighed, and used for further analysis.

### Statistical analysis

Values are presented as the mean ± SEM of at least three independent experiments. Data were analyzed using one way analysis of variance (ANOVA) and Tukey’s multiple comparison tests using GraphPad prism software version 5.01 (La Jolla, CA, USA). Statistical significance was considered at p<0.05.

## SUPPLEMENTARY MATERIALS FIGURES



## Abbreviations

3**-**MA: 3-Methyladenine
AF-1: Activation Function-1
Akt: Protein Kinase B
AMPK: 5’ Adenosine monophosphate-activated protein kinase
EGF: Epidermal Growth Factor
ER: Estrogen receptor
ERα: Estrogen receptor alpha
ERβ: Estrogen receptor beta
ERE: Estrogen Response Elements
ERK: Extracellular Regulated Kinase
FoxO3A: Forkhead box O3A
GPER: G Protein-Coupled Estrogen Receptorf
IGF**-1**: Insulin-like Growth Factor-1
JAK: Janus Kinase
JNK: c-Jun N-terminal Kinase
MAPK: Mitogen Activated Protein Kinase
MTS: 3-(4,5-dimethylthiazol-2-yl)-5-(3-carboxymethoxyphenyl)-2-(4-sulfopheny)-2H-tetrazolium
PCNA: Proliferating Cell Nuclear Antigen
PI3K: Phosphoinositide 3-Kinase
STAT3: Signal Transducer and Activator of Transcription 3
ROS: Reactive Oxygen Species
