# Supplementary material for: Estrogen receptor signaling mediates leptin-induced growth of breast cancer cells via autophagy induction
Source: Oncotarget. 2017 Nov 25;8(65):109417–35. doi: 10.18632/oncotarget.22684 (PMC5752531; doi:10.18632/oncotarget.22684)
Supplement: Supplementary file 1 [file oncotarget-08-109417-s001.pdf]

Estrogen receptor signaling mediates leptin-induced growth of breast cancer cells via autophagy induction

SUPPLEMENTARY MATERIALS

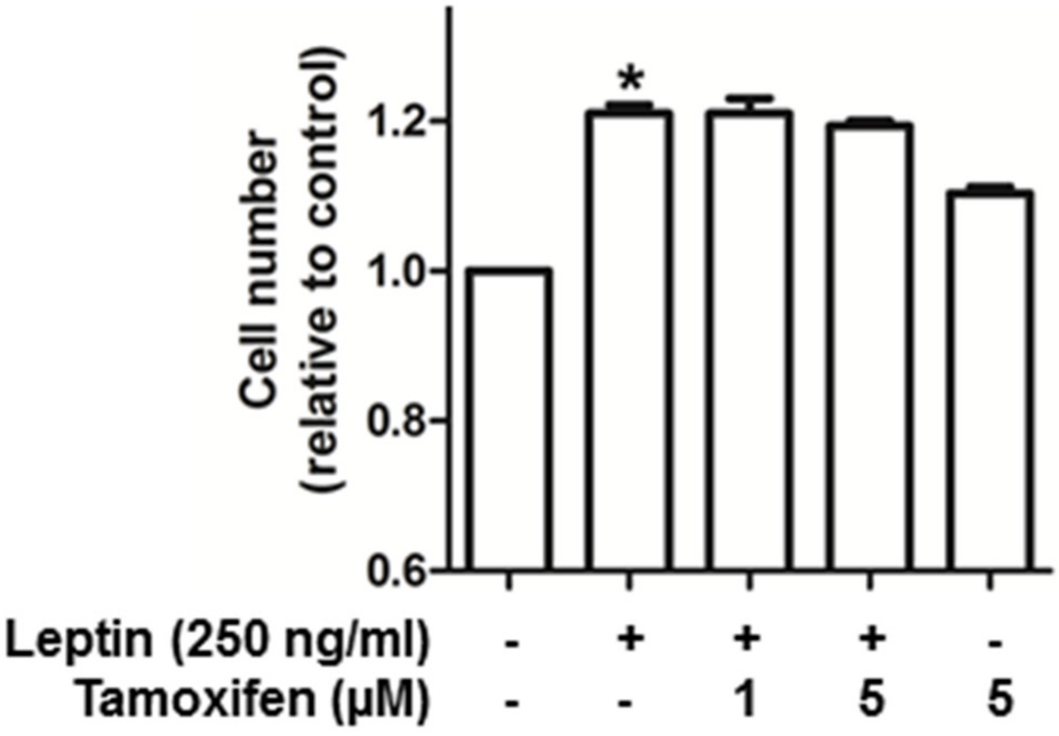

Supplementary Figure 1: Effect of tamoxifen on leptin-induced increased cell viability in HepG2 cells. HepG2 cells were pretreated with indicated concentrations of tamoxifen for 1 h followed by treatment with leptin for an additional 48 h. Cell viability was assessed by MTS assay. Values are presented as mean ± SEM (n=3). * denotes $P < 0.05$ compared with control cells.

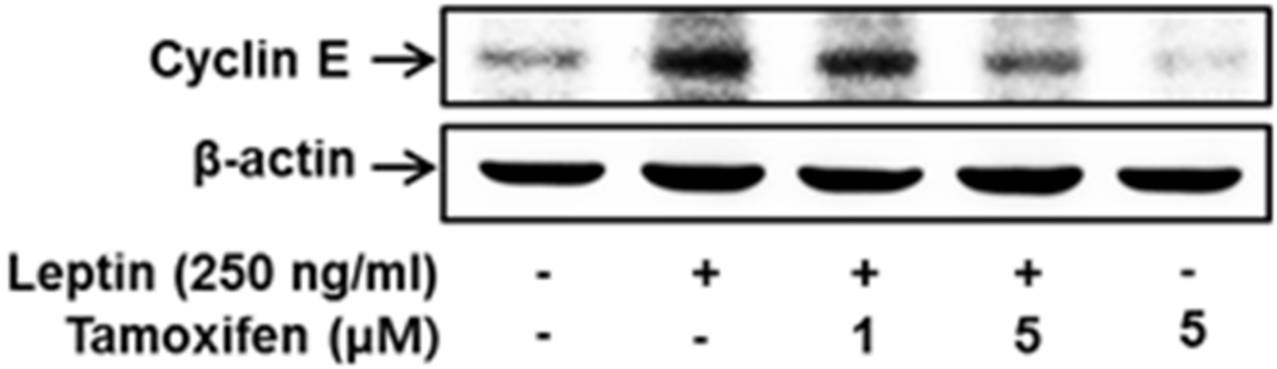

Supplementary Figure 2: Effect of tamoxifen on leptin-induced cyclin E expression in MCF-7 cells. MCF-7 cells were pretreated with indicated concentrations of tamoxifen for 1 h. Cells were further stimulated with leptin (250 ng/mL). Cyclin E protein expression level was determined by Western blot analysis. Representative images from three independent experiments are shown.

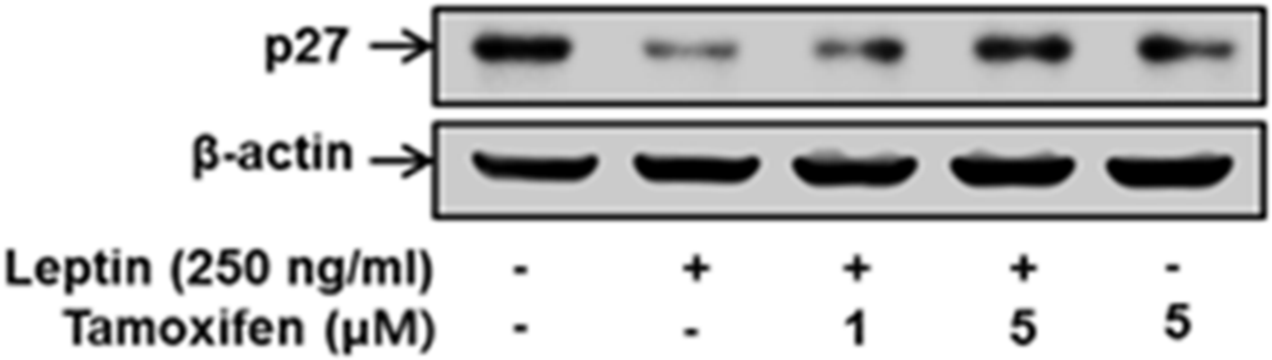

Supplementary Figure 3: Effects of tamoxifen on the modulation of p27 expression induced by leptin in MCF-7 cells. MCF-7 cells were pretreated with indicated concentrations of tamoxifen for 1 h. Cells were further stimulated with leptin (250 ng/mL). Protein expression level of p27 was determined by Western blot analysis. Representative images from three independent experiments are shown.

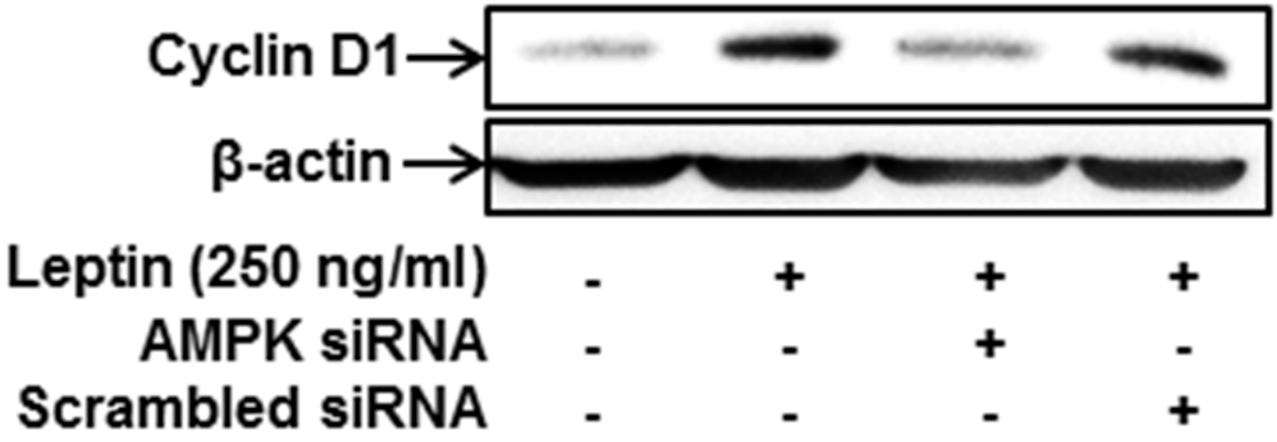

Supplementary Figure 4: Role of AMPK signaling in leptin-induced cyclin D1 expression. MCF-7 cells were transfected with siRNA targeting AMPK for 48 h followed by treatment with leptin additional 48 h. Cyclin D1 protein level was determined by Western blot analysis. Representative images from three independent experiments are shown.

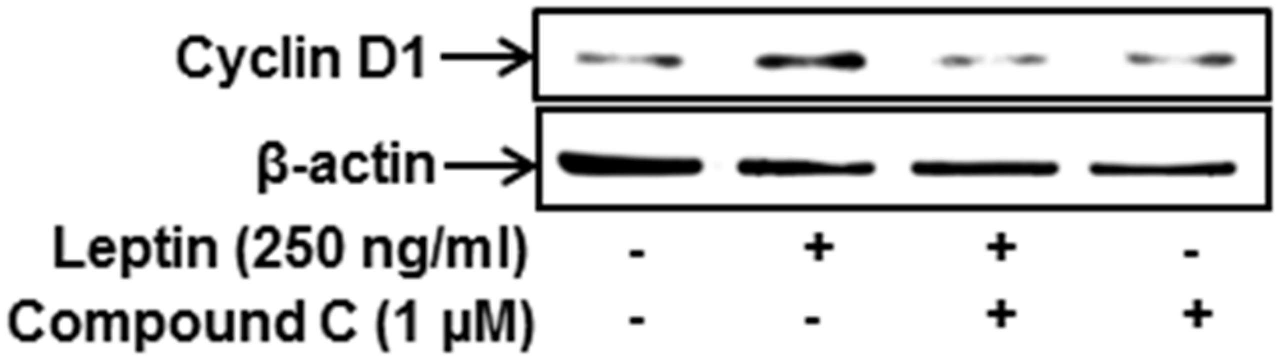

Supplementary Figure 5: Effect of compound C on leptin-induced cyclin D1 expression. MCF-7 cells were pretreated Compound C for 2h followed by treatment with leptin additional 48 h. Cyclin D1 protein level was determined by Western blot analysis. Representative images from three independent experiments are shown.
